# Small Cell Lung Carcinoma: Current Diagnosis, Biomarkers, and Treatment Options with Future Perspectives

**DOI:** 10.3390/biomedicines11071982

**Published:** 2023-07-13

**Authors:** Kristina Krpina, Semir Vranić, Krešimir Tomić, Miroslav Samaržija, Lara Batičić

**Affiliations:** 1 Clinic for Respiratory Diseases Jordanovac, University Hospital Centre Zagreb, 10000 Zagreb, Croatia; kristina.krpina@kbc-zagreb.hr (K.K.); miroslav.samarzija@kbc-zagreb.hr (M.S.); 2 College of Medicine, QU Health, Qatar University, Doha 2713, Qatar; svranic@anubih.ba; 3 Department of Oncology, University Clinical Hospital Mostar, 88000 Mostar, Bosnia and Herzegovina; kresotomic3@gmail.com; 4 Department of Medical Chemistry, Biochemistry and Clinical Chemistry, Faculty of Medicine, University of Rijeka, 51000 Rijeka, Croatia

**Keywords:** small cell lung carcinoma (SCLC), biomarkers, diagnosis, therapeutic targets

## Abstract

Small cell lung cancer (SCLC) is an aggressive malignancy characterized by rapid proliferation, early dissemination, acquired therapy resistance, and poor prognosis. Early diagnosis of SCLC is crucial since most patients present with advanced/metastatic disease, limiting the potential for curative treatment. While SCLC exhibits initial responsiveness to chemotherapy and radiotherapy, treatment resistance commonly emerges, leading to a five-year overall survival rate of up to 10%. New effective biomarkers, early detection, and advancements in therapeutic strategies are crucial for improving survival rates and reducing the impact of this devastating disease. This review aims to comprehensively summarize current knowledge on diagnostic options, well-known and emerging biomarkers, and SCLC treatment strategies and discuss future perspectives on this aggressive malignancy.

## 1. Introduction

Lung cancer remains a significant global health concern, with staggering mortality rates. According to GLOBOCAN, it accounted for 2.1 million new cases and 1.8 million deaths in 2018, making it the leading cause of cancer-related deaths worldwide [1]. Lung cancer is categorized into two main histological types: non-small cell lung cancer (NSCLC) and small cell lung cancer (SCLC). NSCLC comprises approximately 85% of cases, while SCLC represents around 15% [2]. SCLC is an aggressive neoplasm characterized by rapid proliferation, early dissemination, metastases, acquired therapy resistance, and poor outcomes [3]. Each year, approximately 250,000 new cases of SCLC are reported, resulting in at least 200,000 deaths worldwide [1]. While historically more common in men, the prevalence of SCLC among women has risen due to global smoking trends. Exposure to tobacco carcinogens (polycyclic aromatic hydrocarbons and tobacco-specific nitrosamines) is considered a key risk factor for SCLC, as only 2% of all SCLC cases are among never-smokers [4]. Early diagnosis of SCLC is crucial as most patients present with metastatic disease, limiting the potential for curative treatment. While SCLC exhibits initial responsiveness to chemotherapy and radiotherapy, treatment resistance often emerges, leading to a five-year overall survival rate of only 10% [5]. Poor prognosis is associated with factors such as male gender, poor performance status, and age over 70 [5,6]. Diagnostic procedures for SCLC typically involve physical examination, performance status evaluation, laboratory tests, and imaging techniques, including contrast-enhanced CT scans of the chest and abdomen, brain MRI or CT, and optional FDG PET/CT for limited-stage disease. Pathological examination following bronchoscopy, lymph node biopsy, and metastatic lesion biopsy is essential for accurately classifying SCLC [5]. To combat the high mortality rates associated with lung cancer, smoking cessation, and prevention remain the most critical interventions in reducing lung cancer mortality [5,6].

Common clinical manifestations of SCLC at diagnosis include central tumor masses, mediastinal involvement, and extrathoracic spread in 75–80% of patients [6]. Symptoms may include cough, wheezing, dyspnea, hemoptysis, weight loss, pain, fatigue, and paraneoplastic syndromes. Metastasis frequently occurs in the brain, liver, adrenal glands, bone, and bone marrow, often resulting in neurological deficits and paraneoplastic syndromes [7].

With the addition of programmed cell death protein-1 (PD-1) and programmed death-ligand 1 (PD-L1) inhibitors to chemotherapy in the first line of extensive small cell lung cancer (SCLC), a step forward has been made in improving overall treatment outcomes for patients with SCLC [8]. In routine clinical practice, there are currently no available predictive biomarkers for immunotherapy response, and the use of programmed death-ligand 1 (PD-L1) and tumor mutational burden (TMB) testing is not recommended [5]. The need for biomarkers to predict treatment response in patients with SCLC is urgent. Potential biomarkers such as PD-L1 expression, high TMB (TMB-H), and microsatellite instability (MSI-H) need further investigation for applicability in SCLC. Effective biomarkers, early detection, and advancements in therapeutic strategies are crucial for improving survival rates and reducing the impact of this devastating disease. Therefore, this review aims to comprehensively summarize current diagnostic options, well-known and emerging biomarkers, and treatment options of SCLC and discuss future perspectives of this distinct oncological challenge.

## 2. Pathology of SCLC

SCLC belongs to the spectrum of neuroendocrine pulmonary neoplasms that share some common morphologic, ultrastructural, immunohistochemical, and molecular genomic characteristics [9,10]. Four major neuroendocrine pulmonary neoplasms are carcinoids (typical and atypical) and neuroendocrine carcinomas (SCLC and large cell neuroendocrine carcinomas; LCNEC). A typical carcinoid is a low-grade neoplasm, and atypical carcinoid is intermediate-grade, whereas both neuroendocrine carcinomas are, per definition, high-grade neoplasms. The current evidence suggests that carcinoids (typical and atypical) are closely related and etiologically different from SCLC and LCNEC [9,10]. Carcinoids are not precursor lesions of neuroendocrine carcinomas (SCLC and LCNEC) and may be seen more frequently among non-smokers [9,10]. A small subset of carcinoids can be seen in patients with multiple endocrine neoplasia 1 (MEN1) syndrome (OMIM#131100), while somatic *MEN1* gene mutations are commonly observed in carcinoids [9,10]. Rare cases of histologic transformation of epidermal growth factor receptor (EGFR)—or anaplastic large kinase (ALK)-altered pulmonary adenocarcinomas have also been well-documented [11]. It is widely accepted that SCLC has the same endodermal origins as other major subtypes of lung carcinoma (e.g., adenocarcinoma or squamous cell carcinoma), arising from multipotent precursor cells [9,10,12,13].

**Figure 1:**
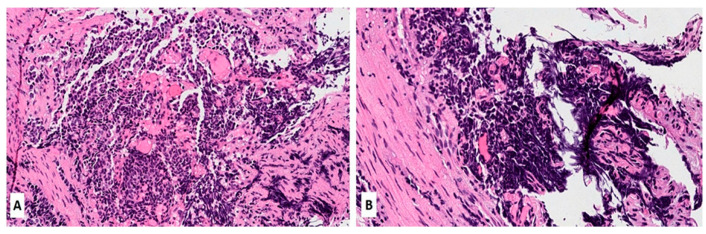
(**A**,**B**) Hematoxylin and Eosin (H&E) stain of a lung biopsy showing a small cell carcinoma with sheet-like diffuse growth pattern and basophilic appearance (A, magnification 10×); Image 1B reveals a prominent nuclear molding of neoplastic cells (magnification 20×).

Morphologically, SCLC is composed of densely packed, small neoplastic cells with scanty cytoplasm and finely granular nuclear chromatin but without prominent nucleoli; nuclear molding and smudging are commonly present (Figure 1A,B). The cells are round or oval, although spindle cells (fusiform pattern of cancer cells) are frequently seen. Mitotic figures are numerous, while the tumor necrosis and crush artifacts may be extensive.

SCLC expresses neuroendocrine markers, such as synaptophysin, chromogranin-A, and CD56/NCAM, which should be used as a panel [14,15]. CD56 is the most sensitive as it stains 90–100% of all SCLC, while synaptophysin and chromogranin-A can be negative in >50% of cases [16,17,18]. Neuron-specific enolase (NSE) is frequently positive in SCLC but is considered non-specific due to its widespread expression in non-neuroendocrine neoplasms (both pulmonary and extrapulmonary) [19]. Thyroid transcription factor 1 (TTF-1) is positive in ~80–90% of cases [10]. Other pulmonary biomarkers, including Napsin-A (positive in adenocarcinomas), p63, and p40 (positive in squamous cell carcinomas), are not immunoreactive in SCLC and can help in differential diagnosis, particularly on small biopsies. Other challenging cases (metastatic neuroendocrine tumors from other anatomic locations, e.g., mammary, gastrointestinal, or Merkel cell carcinoma from the skin) can be resolved using clinical history and other specific immunohistochemical biomarkers.

## 3. Genomic Features of SCLC

Recent research has focused on understanding the genetic basis of SCLC to identify new therapeutic targets and develop more effective treatments [20]. Genetic alterations contribute significantly to the development and progression of SCLC. Concomitant inactivation of two tumor suppressor genes, *TP53* and *RB1,* is found in most SCLC cases [21,22] and is found in up to 90% and 50–90% of SCLC cases, respectively. These molecular features are strikingly different from those seen in NSCLC, in which various oncogenic driver mutations/fusions prevail (e.g., *EGFR, KRAS, ALK, BRAF, RET, ROS1, MET, NTRK1-3, HER2/ERBB2*) [22,23]. Additionally, genetic alterations contributing to SCLC’s development include amplifying the MYC family of oncogenes (*MYC, MYCL*, and *MYCN*), inactivation of the phosphatase and tensin homolog (*PTEN*) tumor suppressor gene, and mutations in the Notch signaling pathway. Genomic alterations of *MYC* family members are seen in SCLC and represent biomarkers of poor prognosis. In particular, *MYCN* alterations are related to SCLC cases with immunotherapy failure. The most important genes altered in SCLC in humans are summarized in Table 1.

Different studies have identified recurrent mutations in chromatin remodeling genes, such as *ARID1A, ARID1B,* and *SMARCA4*, which regulate gene expression. These mutations may contribute to the dysregulation of critical genes involved in cell proliferation and survival, leading to the development of SCLC, characterized by a high frequency of mutations in genes that regulate cell cycle and DNA damage response pathways, such as *TP53, RB1*, and *PTEN*. Additionally, SCLC often exhibits widespread chromosomal instability, with frequent amplifications and deletions of large genome regions. In addition to these genetic alterations, SCLC is characterized by a high frequency of copy number alterations, including amplification of MYC family members and deletion of the tumor suppressor gene cyclin-dependent kinase inhibitor 2A (*CDKN2A*) [24]. In addition, the changes in the stroma and immune microenvironment are additional factors involved in the pathogenesis of SCLC [25].

Overall, the genetic landscape of SCLC is complex and heterogeneous, with multiple genetic alterations contributing to its aggressive phenotype. Understanding the underlying genetic mechanisms of SCLC is crucial for developing effective targeted therapies and personalized treatment strategies for patients with this aggressive cancer. SCLC mutational characteristics reveal a clear causal connection with smoking. Direct scientific evidence confirms that carcinogens from tobacco are responsible for initiating SCLC [26].

Genomic profiling in patients with SCLC has not revealed mutationally defined subtypes of SCLC. However, due to the lack of larger studies, this may be a consequence of the insufficient number of tumor samples included in analyses. Therefore, there is a substantial need for clinical trials that include the analyses of tumor tissue to identify vital genomic triggers. However, there is an accentuated difficulty in tumor material collection. Ethnicity or smoking status did not affect the consistency of mutational differences; however, the prevalence of oncogenic triggers is considered higher in never-smokers with SCLC compared to tobacco users [27].

In addition, genetically modified mice have provided critical genetic lessons and contributed to the knowledge of molecular mechanisms of SCLC etiopathogenesis, metastasis, and response to treatment. It has been shown that tumors in mice show genetic alterations and histological features like those in humans. Ferone et al. provided a comprehensive review of lung cancers and lessons from mouse studies, showing an enormous contribution of animal studies in pulmooncology [28].

**Table 1:** Most important genes altered in SCLC (mostly according to memorial Sloan Kettering-integrated mutation profiling of actionable cancer targets—MSK-IMPACT sequencing of SCLC tumors)—data adopted from Cheng et al. [29], Rudin et al. [23], and Liu et al. [30].

Gene	Aliases	Gene Location on Human Chromosome and Number of Amino Acids	Gene Alteration in SCLC	Known Function and Features	Frequency of Mutation in SCLC (% in Various Cohorts)	Refs.
*TP53*	Tumor protein 53; *p53*; Phosphoprotein P53; Antigen NY-CO-13; Transformation-Related Protein 53; *BCC7*, *LFS1*, *TRP53*, tumor protein *BMFS5*	Chromosome 17 at position 17p13.1.; 375 amino acids	Inactivating mutation; deletion	Nuclear phosphoprotein involved in the regulation of cell proliferation; tumor suppressor; transcription regulation	77–89	Chang et al. [31] Rudin et al. [23]
*RB1*	*RB1*, *pRb*, *RB*, retinoblastoma 1, *OSRC*, *PPP1R130*, *p105-Rb*, *pp110*, Retinoblastoma protein, *RB* transcriptional corepressor 1, *p110-RB1*	Chromosome 13 at position 13q14.1-q14.2.; 928 amino acids	Inactivating mutation; deletion; loss or inactivation of both copies of the gene	Tumor suppressor protein that is dysfunctional in several major cancers. Prevents excessive cell growth by inhibiting cell cycle progression -key regulator of the G1/S transition of the cell cycle	50–90	George et al. [21] Febres-Aldana et al. [32]
*KMT2D*	*KMT2D*, *ALR*, *KABUK1*, *MLL2*, *MLL4*, lysine methyltransferase 2D, histone-lysine methyltransferase 2D, *TNRC21*, *AAD10*, *KMS*, *CAGL114*	Chromosome 12 at position 12q13.12.; 5316 amino acids	Inactivating mutation; deletion; gene fusion; truncating nonsense/frameshift/splice site mutations	Key regulator of transcriptional enhancer function; major enhancer regulator in mammalian cells, including regulation of development, differentiation, metabolism, and tumor suppression.	5–13	Wu et al. [33] Simbolo et al. [34] Augert et al. [35]
*CREBBP*	*AW558298*, *CBP*, *CBP/p300*, *KAT3A*, *p300/CBP*, *RSTS*, *CREB* binding protein, *RSTS1*, *MKHK1*	Chromosome 16 at position 16p13.3. 2414 amino acids.	Inactivating mutation, deletion	Crucial role in transcriptional regulation and chromatin remodeling. Interacts with various transcription factors and coactivators, influencing the expression of target genes involved in cell growth, differentiation, and development.	4–10	Carazo et al. [36] Jia et al. [37]
*PTEN*	*PTEN*, *10q23del*, *BZS*, *CWS1*, *DEC*, *GLM2*, *MHAM*, *MMAC1*, *PTEN1*, *TEP1*, phosphatase and tensin homolog, Phosphatase and tensin homolog, *PTENbeta*	Chromosome 10 at position 10q23.3. 403 amino acids	inactivating mutations, deletions, or loss of expression	Tumor suppressor involved in the regulation of the *PI3K/AKT/mTOR* pathway, which plays a critical role in cell survival and proliferation. PTEN’s protein phosphatase activity may be involved in the regulation of the Cell cycle, preventing cells from growing and dividing too rapidly.	3–10	Sivakumar et al. [38] Zhang et al. [39]
*FAT1*	*CDHF7*, *CDHR8*, *FAT*, *ME5*, *hFat1*, *FAT* atypical cadherin 1	Chromosome 4 at position 4q35.2. 4410 amino acids	Inactivation mutation; deletion	Cell-cell adhesion, migration and communication, regulation of tissue growth, cell polarity, and migration; tumor suppressor gene	2–10	JiaXin et al. [40] Pop-Bica et al. [41]
*PIK3CA*	*PIK3CA*, *CLOVE*, *CWS5*, *MCAP*, *MCM*, *MCMTC*, *PI3K*, *p110-alpha*, *PI3K-alpha*, phosphatidylinositol-4,5-bisphosphate 3-kinase catalytic subunit alpha, *CLAPO*, *CCM4*	Chromosome 3 at position 3q26.3.; 1068 amino acids	Activating mutation; mutations in specific regions	The *PIK3CA* gene for synthesis of the catalytic subunit alpha of the enzyme phosphatidylinositol 3-kinase, having crucial role in cell growth, proliferation, and survival	1–7	Hung et al. [42] Pop-Bica et al. [41]
*NOTCH1*	*NOTCH1*, *Notch1*, *9930111A19Rik*, *Mis6*, *N1*, *Tan1*, *lin-12*, *AOS5*, *AOVD1*, *hN1*	Chromosome 9 at position 9q34.3. 2527 amino acids	Inactivating mutation	Tumor suppressor; involved in cell signalling processes	1–6	Li et al. [43] Roper et al. [44] Herbreteau et al. [45]
*NF1*	*NFNS*, *VRNF*, *WSS*, neurofibromin 1	Chromosome 17 at position 17q11.2. 2818 amino acids	Inactivating mutation, deletion	Tumor suppressor. Neurofibromin 1 plays a role in regulating cell growth and proliferation by negatively regulating the activity of Ras, associated with uncontrolled cell growth.	3–4	Ross et al. [46] Shimizu et al. [47]
*APC*	*BTPS2*, *DP2*, *DP2.5*, *DP3*, *GS*, *PPP1R46*, adenomatous polyposis coli, *WNT* signaling pathway regulator	Chromosome 5 at position 5q22.2. 2843 amino acids	Inactivating mutation, deletion	Crucial role in regulating the Wnt signaling pathway and controlling cell proliferation, growth, differentiation, and migration.	3–4	Jin et al. [48] Grote et al. [49]
*EGFR*	*ERBB*, *ERBB1*, *HER1*, *NISBD2*, *PIG61*, *mENA*, epidermal growth factor receptor, *erbB-1*, *ERRP*	Chromosome 7 at position 7p12.1. 1210 amino acids	Activating mutation	Oncogene; a receptor tyrosine kinase that plays a critical role in cell growth, proliferation, and survival; involved in *RAS* signaling pathway.	3–4	Ding et al. [50] Hao et al. [51]
*KRAS*	*C-K-RAS*, *CFC2*, *K-RAS2A*, *K-RAS2B*, *K-RAS4A*, *K-RAS4B*, *KI-RAS*, *KRAS1*, *KRAS2*, *NS*, *NS3*, *RALD*, *RASK2*, *K-ras*, *KRAS* proto-oncogene, *GTPase*, *c-Ki-ras2*, *OES*, *c-Ki-ras*, *K-Ras 2*, *K-Ras*, Kirsten Rat Sarcoma virus	Chromosome 12 at position 12p12.1. 189 amino acids	Activating mutation	A GTPase involved in cell signalingpathways that regulate cell growth and proliferation (*RAS*/MAPK). *KRAS* mutations can lead to the constitutive activation of the KRAS protein, resulting in dysregulated cell signaling and increased cell proliferation.	1–3	Otegui et al. [52] Li et al. [53]
*NOTCH3*	*CADASIL*, *CASIL*, *IMF2*, *LMNS*, *CADASIL1*, notch 3, notch receptor 3	Chromosome 19 at position 19p13.2. 2345 amino acids	Inactivating mutation, deletion	Involved in cell signaling pathways. Notch signaling plays a critical role in cellular processes, such as cell fate determination, differentiation, and development.	<3	Herbreteau et al. [45] Du et al. [54]
*ARID1A*	*B120*, *BAF250*, *BAF250a*, *BM029*, *C1orf4*, *ELD*, *MRD14*, *OSA1*, *P270*, *SMARCF1*, *hELD*, *hOSA1*, *CSS2*, AT-rich interaction domain 1A	Chromosome 1 at position 1p36.11. 2254 amino acids	Inactivating mutation, deletion	Tumor suppressor gene; plays a crucial role in regulating chromatin remodeling and gene expression; involved in various cellular processes, including DNA repair, cell cycle regulation, and differentiation.	<3	Du et al. [54] Devarakonda et al. [55]
PTPRD	HPTP, HPTPD, HPTPDELTA, PTPD, RPTPDELTA, protein tyrosine phosphatase, receptor type D, protein tyrosine phosphatase receptor type D, R-PTP-delta	Chromosome 9 at position 9p23.3. 1840 amino acids	Inactivating mutation, deletion	Protein tyrosine phosphatase receptor that plays a role in regulating cell signaling pathways, including those involved in cell growth, differentiation, and migration.	<3	Sato et al. [56]
ATRX	ATR2, JMS, MRXHF1, RAD54, RAD54L, SFM1, SHS, XH2, XNP, ZNF-HX, MRX52, alpha thalassemia/mental retardation syndrome X-linked, chromatin remodeler, ATRX chromatin remodeler	X chromosome at position Xq21.1.	Inactivating mutation, deletion	Tumor suppressor; plays a critical role in chromatin remodeling and the regulation of gene expression. ATRX is involved in maintaining the stability and structure of telomeres and in cell signaling	<2	Du et al. [54]

## 4. Biomarkers in SCLC

In contrast to NSCLC, the discovery of therapeutic targets in SCLC has not been easy, partly because driver mutations are in first-line loss of function or untargetable, e.g., MYC family members [23]. The recent division of SCLC into molecular subtypes based on the expression of transcription factors has provided an essential step in searching for new therapeutic targets for the disease. This classification system identifies four distinct subtypes of SCLC: achaete-scute homolog 1 (ASCL1), neurogenic differentiation factor 1 (NEUROD1), yes-associated protein 1 (YAP1), and POU class 2 homeobox 3 (POU2F3) [57].

New blood-based biomarkers for the early detection of lung cancer have been developed and evaluated, with several showing promising results. “Liquid biopsy”—biomarkers such as tumor-derived extracellular vesicles, circulating tumor cells (CTC), and circulating tumor DNA (ctDNA) seem to be promising tools in cancer monitoring. For example, SCLC cells express different tumor-specific markers, including Delta-like protein 3 (DLL-3), which may be associated with a worse prognosis in patients with SCLC [58]. However, whether these biomarkers listed in Table 2 will impact cancer control in the population, especially in cancer with aggressive biologic behavior such as SCLC, remains unknown. To date, it seems that patients with SCLC have the greatest number of CTC, which was suggested to be a prognostic biomarker for clinically evaluating therapy efficacy [59]. Likewise, CTC-derived DNA and plasma cell-free DNA, along with their genomic alterations, have been recognized as potential non-invasive biomarkers that could provide insights into treatment efficacy and the occurrence of SCLC relapse [60].

Furthermore, the characterization of extracellular vesicles, such as exosomes, appears to be a promising tool and alternative source for various analytes in liquid biopsies [61]. This approach has the potential to significantly contribute to the identification of new biomarkers for the diagnosis and monitoring of SCLC patients, as well as the development of promising prognostic models. Emerging predictive and prognostic biomarkers are crucial and indispensable for selecting the most suitable therapeutic option for patients with SCLC.

To date, genetic alterations of *MYC* were noticed in about 20% of patients with SCLC, representing the third most common genetic abnormality following *TP53* and *RB1* and a potential biomarker of targeted therapy [62]. PD-L1, TMB, and MSI-H have been studied as potential predictive biomarkers for response to immune checkpoint inhibitors (ICIs) in patients with SCLC [62]. Schlafen 11 (SLFN11) is a DNA/RNA helicase that sensitizes cancer cells to DNA-damaging agents. The newest scientific evidence confirms its importance as a promising predictive biomarker for several therapeutics, including platinum and PARP inhibitors [63]. Expression of SLFN11 in CTCs provides a potential biomarker of sensitivity for DNA-damaging chemotherapy drugs and poly (ADP-ribose) polymerase (PARP) inhibition in SCLC patients [62]. Therefore, detecting SLFN11 by liquid biopsy in circulating CTCs may provide a valuable non-invasive alternative to tissue sampling [64].

**Table 2:** Potential biomarkers in small cell lung carcinoma.

Biomarker	Type	Potential Application	References
Delta-like ligand 3 DLL3	Tumor-specific marker	Biomarker for SCLC prognosis	Chen et al. [58]
Circulating tumor cells (CTC)	Liquid biopsy biomarker	Prognostic biomarker for therapy evaluation of therapy efficacy	Roumeliotou et al. [59]
Circulating tumor DNA (ctDNA)	Liquid biopsy biomarker	Biomarker for treatment efficacy and relapse detection	Almodovar et al. [60]
Exosomes	Extracellular vesicles	Non-invasive biomarkers for prognosis	Zhang et al. [61]
MYC proto-oncogene/bHLH transcription factor (MYC)	Genetic alteration	Potential biomarker for targeted therapy	Taniguchi et al. [62]
Programmed death-ligand 1 (PD-L1)	Immune checkpoint protein	Potential biomarker for immunotherapy response	Taniguchi et al. [62]
Tumor mutational burden (TMB)	Mutation load of a tumor	Potential biomarker for immunotherapy response	Taniguchi et al. [62] and Li et al. [65]
Microsatellite instability (MSI-H)	Genetic marker of Microsatellite Instability	Potential biomarker for immunotherapy response	Taniguchi et al. [62] and Chang et al. [66]
Schlafen 11 (SLFN11)	Liquid biopsy biomarker	Potential biomarker for the response on DNA damaging chemotherapy and PARP inhibition	Taniguchi et al. [62] and Zhang et al. [63]

### 4.1. Biomarkers of Response to Immune Checkpoint Inhibitors in SCLC

Immunotherapy with ICI has markedly improved the treatment of various solid and hematologic malignancies, including lung cancer. ICIs target PD-1 and its ligand (PD-L1). Validated predictive biomarkers associated with a response to ICI include PD-L1 expression (in tumor or immune cells), TMB-H, and MSI-H status. Several ICIs have been approved for NSCLC, while for SCLC, two ICI (nivolumab and atezolizumab) obtained approvals from FDA in 2018 and 2019, respectively [67,68]. In contrast to NSCLC, no predictive biomarkers to ICI response (or resistance) in SCLC have been validated and approved. However, several studies confirmed a substantial therapeutic benefit of adding ICIs to conventional chemotherapy as a first-line treatment for extensive SCLC [69,70]. Facchinetti et al. summarized the results of four randomized trials involving >1500 patients with SCLC treated with combined ICI/chemotherapy vs. chemotherapy alone (platinum–etoposide). They found a slight (10%) but clinically significant improvement in survival outcomes of SCLC patients treated with a combination of chemotherapy and ICI [69]. The authors also highlighted an unmet need for proper predictive biomarkers for ICI. Findings from another systematic review conducted by Zhou et al. also supported the use of the combined treatment with ICI (durvalumab and atezolizumab) and etoposide-based chemotherapy as an optimal first-line treatment approach for patients with extensive-stage SCLC [71]. Chen et al. summarized the results of four clinical trials (>1500 patients with extensive stage SCLC, ES-SCLC), focusing on the efficacy of four different ICIs as a first-line treatment (atezolizumab, pembrolizumab, nivolumab, and durvalumab). They found that none of the ICIs was superior regarding overall and disease-free survival. However, durvalumab was superior to atezolizumab but with higher toxicity (immune-related adverse effects) [72]. Recently designed and ongoing clinical trials appear to be more promising, including predictive tissue-based testing before and after treatment (e.g., Tempus Sculptor Study) [73].

#### 4.1.1. PD-L1 Expression in SCLC

A recently published systematic review with meta-analysis [74] analyzed PD-L1 expression in ~2800 SCLC samples reported in 27 studies. The overall PD-L1 expression was 26% with a favorable prognostic impact, although it did not reach statistical significance [74]. However, the results were heterogeneous, different cutoffs for the definition of PD-L1 positivity were used, and marked variability in subcellular localization of PD-L1 protein in cancer cells was also observed/assessed. A recently published study by Lang et al. revealed a lower (~10%) PD-L1 positivity in cancer cells. The stromal PD-L1 positivity was observed in ~60% of cases with a significant favorable impact on patients’ outcomes. Notably, the authors did not find a significant correlation between PD-L1 expression and molecular subtypes of SCLC [75]. Yu et al. explored PD-L1 expression in SCLC regarding the anatomic location (central vs. peripheral) and TTF-1 expression (positive vs. negative) [76]. They found a more prevalent PD-L1 expression in centrally located, TTF-1-positive SCLC [76].

In contrast to other studies, they found PD-L1 expression as an adverse prognostic factor in SCLC associated with vascular and lymphatic invasion [76]. In summary, variable PD-L1 expression has been reported in SCLC. However, its predictive value has not yet been established, so routine ICI treatment testing is not recommended.

#### 4.1.2. Tumor Mutational Burden (TMB)

TMB is defined as the number of somatic mutations in cancer per megabase of interrogated genomic sequence [77]. TMB has been validated as a predictive biomarker to ICI in multiple studies, leading to the approval of pembrolizumab for all solid tumors with TMB-H, regardless of histotype (tumor-agnostic approach) [78]. One of the largest studies involving less common solid tumors was conducted by Shao et al. [79]. The study included 305 SCLC samples, of which 37% had TMB-H defined as ≥10 mutations/megabase. The study by Hellmann et al. (2018) involved 401 patients with ES-SCLC, out of which 211 (53%) had comprehensive molecular profiling completed. They found that 27% of tested samples had TMB-H with a significant impact on the therapeutic benefit of ICI, particularly the combination of nivolumab and ipilimumab [80]. Zhou et al. explored a small cohort of SCLC patients using next-generation sequencing (NGS). The median TMB was 21.7 mutations/Mb (range 9.3–55.9), and high TMB (defined as >21 mutations/Mb) was a good prognostic factor of OS [81]. Li et al. (2023) explored a small cohort of SCLC (n = 18), revealing TMB-H in 78% of cases using a threshold of seven mutations/megabase [53]. Based on the current evidence, a substantial proportion of SCLC cases may harbor TMB-H (regardless of the threshold for its definition). This feature may be used for treatment with ICIs.

#### 4.1.3. Microsatellite Instability (MSI-H) in SCLC

Microsatellite instability (MSI-H) is a specific molecular genomic alteration with a hyper-mutable phenotype of a cell caused by the impaired DNA mismatch repair machinery (MMR). MSI-H is particularly common in colorectal cancer, a subset of endometrial, gastric, and upper urinary tract carcinomas, while other cancers are rarely affected by the MSI-H/MMR phenotype. From the clinical point of view, MSI-H/dMMR status predicts response to ICIs and is an approved biomarker for pembrolizumab therapy irrespective of histotype (tumor-agnostic approach) [82].

Merlo et al. published one of the earliest papers reporting MSI status in SCLC [83]. The authors found a frequent MSI-H in primary SCLC. However, later studies did not confirm these observations revealing a rare occurrence of MSI-H in lung cancers, but still higher in SCLC compared with NSCLC (~3% vs. 0.1%) [84]. The same study showed that MSI-H SCLC also had TMB-H [84]. A comprehensive molecular analysis of 21 SCLC cell lines revealed that MMR deficiency does not play a prominent role in the pathogenesis of SCLC [85]. Another study conducted by Yanagawa et al. (2021) revealed a lower (1.1%) prevalence of MSI-H in SCLC samples [86]. A large, comprehensive genomic profiling study of Chinese patients with SCLC revealed no MSI-H among 111 SCLC-tested cases [87]. Based on the available data, SCLC is characterized by a low prevalence of MSI-H; however, rare cases of MSI-H SCLC may also harbor concomitant TMB-H and are more likely to benefit from immunotherapeutic approaches.

#### 4.1.4. Delta-Like Ligand 3 (DLL3)

Delta-like ligand 3 (DLL3) represents an inhibitory ligand of the Notch receptor whose overexpression on the surface of neoplastic neuroendocrine cells is associated with tumor progression [88]. The Notch signaling pathway is a highly conserved pathway involved in various developmental processes, including developing pulmonary neuroendocrine cells [89]. DLL3 expression is regulated by achaete-scute homolog 1 (ASCL1), a transcription factor required to develop pulmonary neuroendocrine cells properly. DLL3 is a powerful oncogenic driver in SCLC [90]. More than 80% of SCLC overexpress DLL3 protein in diffuse and homogenous patterns [89,91]. DLL3 expression has also been described in various extrapulmonary neuroendocrine neoplasms (particularly high-grade carcinomas), such as bladder, cervix, anus, prostate, and bile duct neuroendocrine carcinomas [88,89,92,93]. In contrast, its expression in neuroendocrine carcinomas of the breast was low [94]. DLL3 expression in neuroendocrine neoplasms is associated with tumor progression and poor clinical outcomes [88].

Although initial clinical trials (Phase II TRINITY Study; Phase III TAHOE Study) revealed poor to modest therapeutic effects of DLL3 inhibitor rovalpituzumab tesirine in patients with DLL3-positive SCLC [95,96], recent data with novel DLL3 inhibitors such as tarlatamab appear to be promising [97]. Tarlatamab (TMG 757) is a “first-in-class DLL3-targeted bispecific T-Cell engager” that binds to DLL3 and CD3 receptors, activating T-cell mediated tumor cell lysis. In the Phase I study conducted by Paz-Ares et al. (2023), tarlatamab exhibited encouraging therapeutic effects (objective response rate 23%) and an acceptable safety profile in heavily pretreated SCLC patients. Interestingly, DLL3 expression assessed by immunohistochemistry correlated well with the therapeutic responses [97].

## 5. SCLC Treatment and Approaches to SCLC Therapy

**Figure 2:**
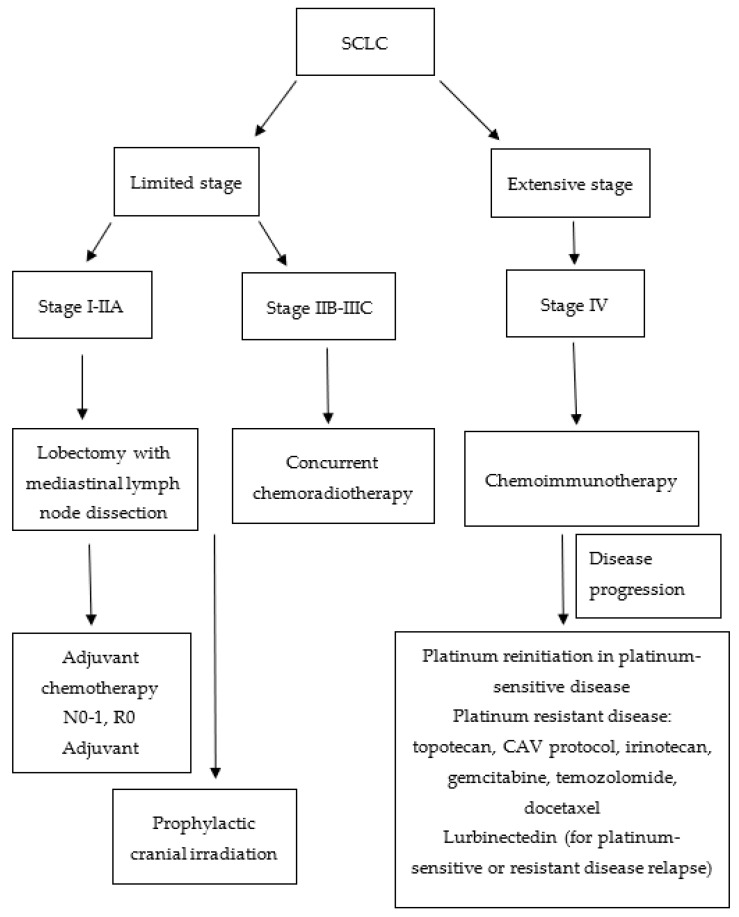
Flowchart of small cell lung cancer (SCLC) treatment algorithm. CAV: cyclophosphamide, doxorubicin, vincristine.

SCLC patient outcomes have not been substantially improved in the era of precision oncology. The treatment algorithm for SCLC is shown in Figure 2. However, systemic therapies, including immunotherapy, show promising results, although many patients do not respond well to the treatment and need alternative or complementary therapeutic approaches. Discrete molecular subcategories of SCLC differ in their responsiveness to a certain therapeutic approach, which opens new questions and directions in the therapies [57]. Landmark studies have been conducted to discover crucial drivers of drug response in cancer cells. One of the largest studies on SCLC cell lines investigated 526 chemical compounds on 63 SCLC cell lines to find promising candidates for new oncological drugs and therapeutic approaches. This extensive study showed that compounds targeting nuclear kinases appear effective in SCLC lines. However, additional investigations, including xenografts, are needed to elucidate their possible therapeutic effectiveness [98]. Many efforts are being invested in screening various compounds to unravel novel targets and biomarkers in SCLC [99]. In addition to synthetic compounds, many phytochemicals and other naturally occurring bioactive compounds like various polyphenols, alkaloids, terpenoids, thiols, and others are intensively investigated to design new anticancer strategies [100]. Different treatment strategies for SCLC, depending on the stage of the disease, are shown in Table 3. Considering the complex treatment algorithm for patients with lung cancer, which encompasses the pathological and molecular phenotype, it is crucial to present each patient to a multidisciplinary team (MDT). The main purpose of the MDT presentation is to facilitate a comprehensive and collaborative approach to the patient’s diagnosis, treatment, and supportive care.

### 5.1. Limited Stage

Surgical treatment of SCLC for stages I-II due to the aggressiveness of the disease and detection of the disease in most cases at an advanced stage is carried out in a small number of patients. Surgical treatment of lobectomy with mediastinal dissection can be performed after extensive staging, in which stage I or II diseases are confirmed. Several retrospective studies have verified a five-year survival rate of 45–65% for stages I-II after operative treatment. For negative mediastinal lymph nodes and margins, adjuvant chemotherapy with cisplatin and etoposide is advised [5,101]. In the case of positive mediastinal lymph nodes or R1 (microscopic residual tumor) and R2 margins (macroscopic residual tumor), chemoradiotherapy is advised. Prophylactic cranial radiotherapy (PCI) is agreed upon with the patient due to conclusive data from the literature, where it is necessary to disclose the benefit and toxicity of PCI.

SCLC is a very chemosensitive tumor with a response rate of 70–90%, and the basis of treatment for most patients in the limited stage of the disease is chemoradiotherapy. The preferred chemotherapy regimen is cisplatin 75 mg/m^2^ day one and etoposide 100 to 120 mg/m^2^ day 1–3 combined with external radiotherapy in a total dose of 45 Gy twice daily (BID). The CONVERT study showed that in combination with chemotherapy, a total radiotherapy dose of 60 Gy once daily is not inferior to 45 Gy BID [70]. By improving disease staging and radiotherapy techniques, the five-year OS of patients treated with chemoradiotherapy was extended from 16% to 34% [102,103]. About 50% of patients have brain metastases, so after the chemoradiotherapy treatment, PCI is performed on the brain area in case of response to the therapy [5]. PCI reduces disease recurrence in the brain and improves three-year overall survival by 5.4% [104]. Due to the greater possibility of cognitive decline, if the patient is older than 70 years or in a worse general condition, ECOG 2 or more PCI is in agreement with the patient.

To improve the treatment of limited SCLC, studies are underway to incorporate immunotherapy with chemoradiotherapy and improve treatment outcomes. For now, immunotherapy and targeted therapy have no role in treating SCLC.

### 5.2. Extensive Stage

For the last 30 years, the mainstay of treatment for extensive SCLC (eSCLC) has been chemotherapy with cisplatin and etoposide, with a median survival of 7–11 months. The addition of immunotherapy with checkpoint inhibitors (CPI), atezolizumab (PD-L1 inhibitor), and durvalumab (PD-L1 inhibitor) in combination with basic platinum chemotherapy achieves a moderate but statistically significant increase in OS for two months, from 10 to 12 months [105,106]. Based on the above results, the basis of the treatment in the first line of eSCLC is chemoimmunotherapy with atezolizumab or durvalumab. After longer clinical follow-up, immunotherapy with atezolizumab or durvalumab can ensure long-term survival at 18 months, 34.0%, and at 36 months, 17.6% in patients with eSCLC [107,108]. New ICIs, such as serplulimab (PD-1 inhibitor) and adebrelimab (PD-L1 inhibitor) combined with chemotherapy, have shown benefit in the first-line treatment of eSCLC.

In contrast, pembrolizumab (PD-1 inhibitor) and ipilimumab (CTLA-4 inhibitor) have not shown benefit in RCTs [109,110,111,112]. In patients in whom immunotherapy is contraindicated or unavailable, the basis of treatment is carboplatin or cisplatin in combination with etoposide [5,101]. In the case of response to chemotherapy, treatment with consolidation radiotherapy of the lung and PCI or MRI brain surveillance is considered [5,101]. The best symptomatic supportive therapy is advised in patients with poor general conditions. In the second line of treatment, several cytostatics are available, and the choice of cytostatic depends on the patient’s general condition, the previous toxicity of the therapy, and the platinum-free interval. In the case of platinum-sensitive disease, reinitiation of platinum is recommended. In the case of platinum-resistant disease, patients can be treated with chemotherapy with a response rate of 15–30%. Topotecan, CAV (cyclophosphamide, doxorubicin, vincristine) protocol, irinotecan, gemcitabine, temozolomide, and docetaxel are used. Recently, lurbinectedin (inhibitor of DNA synthesis) has been approved, based on a good response rate of 35.2% in a phase II study in patients with platinum-sensitive or resistant disease relapse [113].

## 6. New Drugs in the Second Line or Beyond

New emerging therapeutic strategies for SCLC that are under investigation are Aurora A inhibitor, poly (ADP-ribose) polymerase (PARP) inhibitor, ATR kinase inhibitor, CHK1 inhibitor, DLL3 inhibitor, MYC inhibitor, Ganglioside fucosyl-GM1 and inhibitor of the bromodomain (BRD) and extra-terminal domain (BET) family of proteins [114]. Personalization of therapy in a patient with extensive SCLC and DLL3 ligand expression was attempted with rovalpituzumab tesirine, an antibody-drug conjugate (ADC) that destroys tumor cells by intracellular cytostatic deposition. In the Phase 1 study (protocol number NCT01901653), the activity of rovalpituzumab tesirine was evaluated in 82 patients with SCLC who had progressed to one or more lines of therapy. The key finding of the study indicated that 38% of the patients demonstrated a positive response to treatment with rovalpituzumab tesirine in patients with high DLL3 expression, defined as having ≥50% expression on tumor cells [115]. In the Phase II TRINITY study (protocol number: NCT02674568), rovalpituzumab tesirine demonstrated modest clinical activity among the 339 pretreated patients with SCLC, and the response rate for DLL3-positive patients was 13.2% [95]. In a randomized Phase 3 TAHOE study (protocol number: NCT03061812) involving patients with SCLC undergoing second-line therapy, rovalpituzumab tesirine showed inferior OS when compared to topotecan, with a median OS of 6.3 months versus 8.6 months, respectively [96]. The limitations of the Phase 1 and Phase 2 studies with rovalpituzumab tesirine are primarily attributed to their single-arm study designs, lacking a control comparator arm for direct comparison. Furthermore, when compared to the Phase 2 and Phase 3 studies with rovalpituzumab tesirine, the Phase 1 study’s limitations become apparent due to the selective inclusion of patients who may not be representative of the real-world population. For example, one of the key exclusion criteria in the Phase 1 study was the presence of active central nervous system (CNS) metastases [115]. In a different approach to DLL3 receptors, recent data with novel DLL3 inhibitors such as tarlatamab appear promising [97]. The Phase 1 study (protocol number NCT03319940) assessing the efficacy of tarlatamab in heavily pretreated SCLC patients has demonstrated promising results, with a response rate of 23.4% [97]. The Phase 1/2 study (protocol number NCT02446704) evaluating the combination of the PARP inhibitor olaparib with temozolomide in 50 patients with previously treated SCLC revealed a promising new therapeutic strategy, with an overall response rate of 41.7% [116]. In the Phase 2 study (protocol NCT02038647), the combination of the Aurora A kinase inhibitor alisertib with paclitaxel demonstrated efficacy signals in relapsed or refractory SCLC [117]. Additionally, c-Myc expression and mutations were identified as potential predictive biomarkers of alisertib. The disease control rate, defined as the combination of complete response, partial response, and stable disease lasting at least 8 weeks, was significantly higher with alisertib/paclitaxel compared to placebo/paclitaxel (55% versus 33%) in the subgroup of resistant or refractory patients efficacy [118]. Among c-Myc-positive patients, the median progression-free survival was 4.64 months with alisertib/paclitaxel, whereas it was 2.27 months with placebo/paclitaxel. In the phase II study (protocol number NCT02487095), the combination of M6620 (berzosertib), ataxia telangiectasia, and rad3-related inhibitor (ATR), with topotecan in 25 patients with relapsed SCLC, demonstrated a response rate of 36%, successfully meeting the primary efficacy endpoint. Intensive scientific and clinical efforts are being invested in unraveling new therapeutic options for SCLC to provide better responsiveness to this challenging malignancy.

## 7. Conclusions

SCLC represents a significant global health burden with high mortality rates. The discovery of therapeutic targets in SCLC has been challenging compared to NSCLC due to the high prevalence of untargetable driver mutations. Immune checkpoint inhibitors (ICI) combined with chemotherapy have shown significant benefits in treating SCLC. PD-L1 expression, tumor mutational burden, and microsatellite instability are validated predictive biomarkers for ICI response in various cancers, but their predictive value in SCLC remains uncertain. Although several emerging targets have been identified (e.g., DLL3), they failed to provide satisfactory therapeutic benefits. Therefore, further studies are needed to provide and validate novel therapeutic targets and biomarkers for this highly aggressive malignancy.

## Figures and Tables

**Table 3:** Treatment strategies for patients with small cell lung carcinoma.

Stage of the Disease	Treatment Options
Limited	Lobectomy with mediastinal dissection for stage I or II disease after extensive staging
	Adjuvant chemotherapy with cisplatin and etoposide for negative mediastinal lymph nodes and margins
	Chemoradiotherapy for positive mediastinal lymph nodes or R1–R2 margins
	Prophylactic cranial radiotherapy (PCI) in case of response to therapy
	The preferred regimen for chemoradiotherapy: Cisplatin 75 mg/m^2^ day one and etoposide 100 to 120 mg/m^2^ day 1–3
	External radiotherapy in a total dose of 45 Gy twice daily (BID)
	A total radiotherapy dose of 60 Gy once daily is not inferior to 45 Gy BID (CONVERT study)
Extensive-stage: First-line	Chemoimmunotherapy with atezolizumab or durvalumab in combination with platinum-based chemotherapy
	Carboplatin or cisplatin in combination with etoposide
	Consolidation radiotherapy of the lung and prophylactic cranial irradiation (PCI) or MRI brain surveillance if there is a response to chemotherapy
Extensive-stage: Second-line	Platinum reinitiation in platinum-sensitive disease
	Chemotherapy (topotecan, CAV protocol, irinotecan, gemcitabine, temozolomide, docetaxel)
	Lurbinectedin (for platinum-sensitive or resistant disease relapse)
Extensive-stage: Second line or beyond	New emerging therapeutic strategies under investigation (Aurora kinase A inhibitor, poly ADP ribose polymerase (PARP) inhibitor, ataxia telangiectasia, and Rad3 related (ATR) kinase inhibitor, Checkpoint kinase 1 (CHK1) inhibitor, Delta-like ligand 3 (DLL3) inhibitor, MYC inhibitor, Ganglioside fucosyl-GM1, an inhibitor of the bromodomain (BRD) and extra-terminal domain (BET) family of proteins
	Rovalpituzumab tesirine (not proven benefit in phase III randomized controlled trial)
	Tarlatamab (TMG 757)—DLL3-targeted bispecific T-Cell engager
	Olaparib (poly ADP ribose polymerase- PARP inhibitor) in combination with temozolomide
	Aurora kinase inhibitors (positive signals in patients with c-MYC expression SCLC)
	ATR inhibitor in combination with topotecan

## Data Availability

No new data were created or analyzed in this study. Data sharing is not applicable to this article.
